# Engineering of Syndiotactic and Isotactic Polystyrene-Based Copolymers via Stereoselective Catalytic Polymerization

**DOI:** 10.3390/molecules22040594

**Published:** 2017-04-07

**Authors:** Eva Laur, Evgueni Kirillov, Jean-François Carpentier

**Affiliations:** Université de Rennes 1, CNRS, Institut des Sciences Chimiques de Rennes, UMR 6226, F-35042 Rennes CEDEX, France; eva.laur@univ-rennes1.fr

**Keywords:** styrene, syndiotactic polystyrene, isotactic polystyrene, copolymers, stereoselective catalysis, polymerization

## Abstract

This contribution presents an updated overview of the different copolymers containing stereoregular polystyrene blocks. Special emphasis is placed on syndiospecific and isospecific copolymerization of styrene with co-monomers (ethylene and α-olefins, conjugated and non-conjugated dienes, styrene derivatives, etc.). The catalytic systems involved are described and the polymerization mechanisms are discussed. Alternative approaches (simultaneous, living, chain-transfer and graft copolymerization) and the resulting detailed structures and characteristics of the copolymers are also reported.

## 1. Introduction

Production of plastic materials is one of the most important processes of the contemporary chemical industry (some 322 million tons of plastic materials were produced in 2015) and represents an ever-increasing market. Besides commodity polymers, numerous efforts are focused on the development of new generations of materials with more specific properties (engineering and specialty polymers) designed to replace other materials like ceramics or metals. Copolymerization of two (or more) monomers is one of the ways to create new materials, as copolymers have unique properties different from those of the corresponding homopolymers. The copolymerization process is often a better alternative than blending the two homopolymers, generally difficult to obtain, and requiring the use of compatibilizers, especially if the two polymers are chemically different. In addition, the mechanical and physico-chemical properties of copolymers are often unique and can be tuned by varying the ratios of monomers.

Since the discovery of stereoregular polyolefins by Natta in the mid-50s [1,2,3] the synthesis of polymers with controlled microstructures has become an important challenge because it intimately affects their properties such as solubility, crystallinity, and thermal properties. More particularly, syndiotactic polystyrene (sPS) has aroused huge interest since its discovery by Ishihara [4,5,6,7,8,9,10]. Indeed, its high melting point, high solvent and heat resistance, high dielectric constant and low permeability to gases make this material very attractive from an industrial point of view. Isotactic polystyrene (iPS), discovered by Natta before sPS, has been less studied due to its low crystallization rate [11,12,13,14] Compared to iPS, sPS has a much faster crystallization rate (two orders of magnitude higher) [15]. However, the brittleness of sPS and the temperature above 290 °C required for its processing limit its industrial applications. The lack of polar groups in the chain is also an issue regarding its adhesion and compatibility with other materials (metals, ceramics, glasses). The production of new copolymers containing stereoregular polystyrene segments is one of the solutions to tackle the above issues.

Synthesis of stereo/regio-regular copolymers is a major challenge in polymer chemistry. The use of metal complexes, especially group 3 and 4 metallocenes and related derivatives, as (pre)catalysts is arguably the only way to access highly regular polymers, with controlled molecular weight and co-monomer distributions. The development of a new generation of polymerization catalysts is a powerful means to finely tune the microstructure of the polymer, and by extension, the macroscopic properties of the resulting materials [16,17,18,19,20,21,22,23].

**Figure 1:**
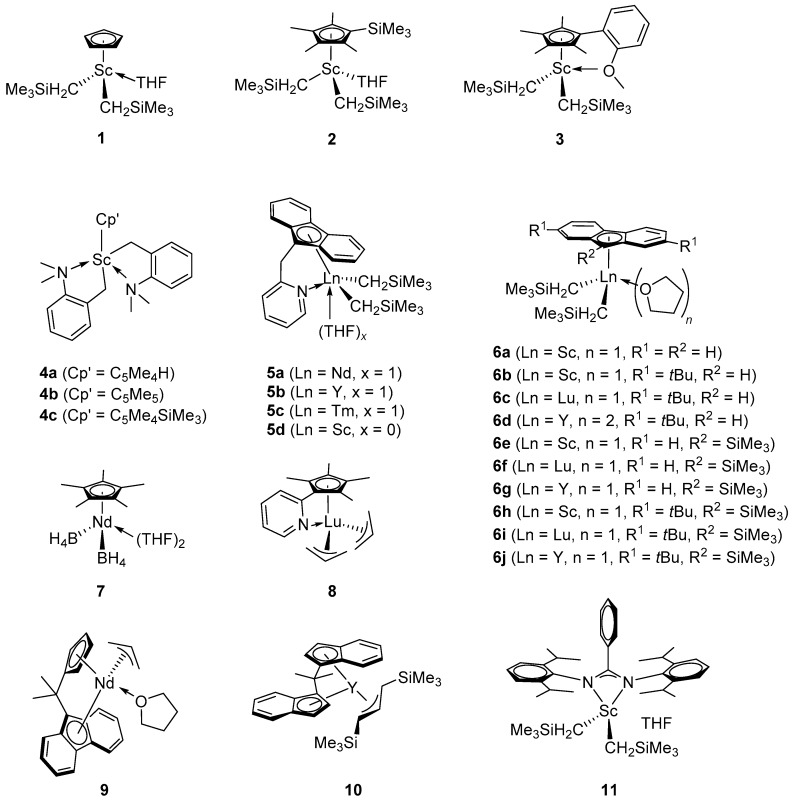
Group 3 metal catalyst precursors used for stereoselective styrene copolymerizations.

**Figure 2:**
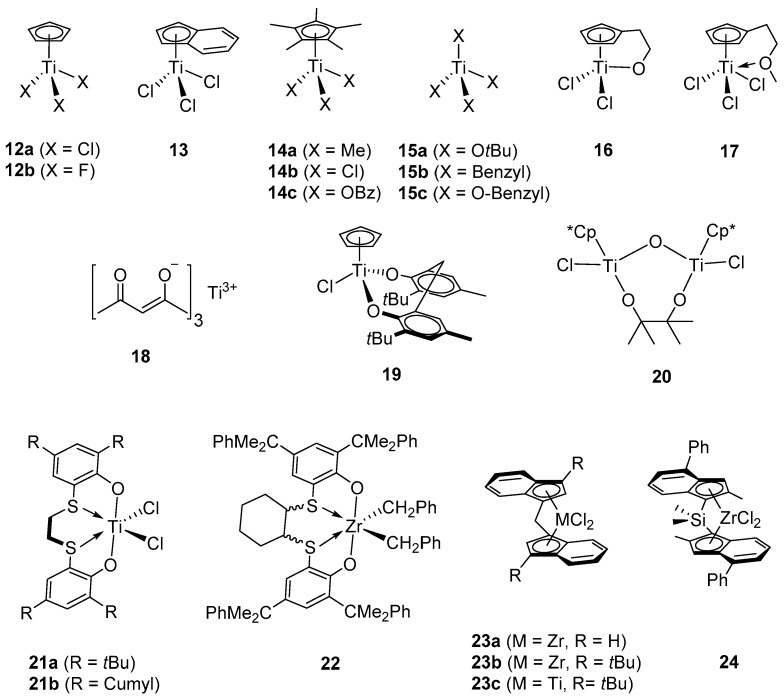
Group 4 metal catalyst precursors used for stereoselective styrene copolymerizations.

In this contribution we present an updated overview of the different copolymers containing stereoregular polystyrene sequences. sPS modification, including syndiospecific copolymerization, have been widely reviewed [24,25]; the present review also includes iPS copolymers and recent progress concerning the synthesis of stereoregular polystyrene copolymers. Different approaches (simultaneous, living, chain-transfer and graft copolymerizations) and resulting detailed structures and characteristics of the copolymers are reported. The catalytic systems involved are also described (Figure 1 and Figure 2), and the polymerization mechanisms are discussed.

## 2. Copolymerization with α-Olefins

**Figure 3:**
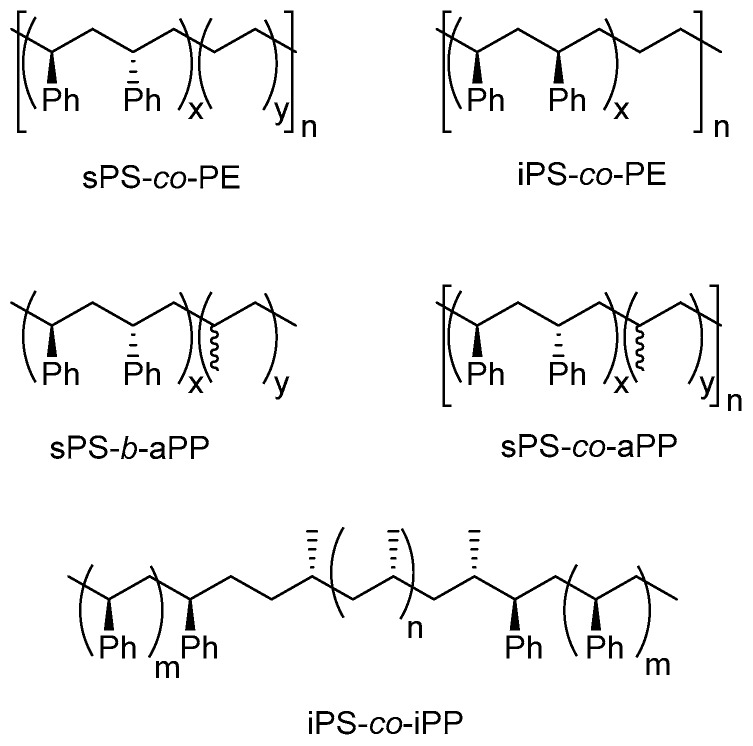
Structures of stereoregular styrene-ethylene and styrene-propylene copolymers.

Copolymerization of styrene with ethylene and, to a lesser extent, α-olefins, has been extensively studied for the past thirty years. Due to the striking differences in reactivity of those monomers, achieving stereoselective copolymerizations remains a quite challenging task. For instance, most of the group 4 catalysts reported for styrene-ethylene copolymerization led to the formation of so-called “ethylene-styrene interpolymers” (ESI), which contain no more than 50% of incorporated styrene and feature no stereoregular microstructure [26]. On the other hand, styrene-propylene copolymerization is even more challenging, because of the conflicting opposite insertion regioselectivity of those two monomers. Styrene-α-olefin copolymerization had widely been reviewed in a previous paper, so the following section is focused on recent progress in this field. The catalytic systems involved and copolymer properties are summarized in Table 1 and Table 2; the copolymer structures are presented in Figure 3.

### 2.1. Ethylene

As mentioned above, many efforts have been focused on the development of catalytic systems for syndiotactic styrene-ethylene copolymers. Upon using traditional syndiospecific titanocenes developed for sPS production, only mixtures of homopolyethylene, homopolystyrene, and styrene-ethylene copolymers without stereoregular polystyrene sequences were obtained [26]. The first syndiotactic styrene-ethylene copolymers were disclosed by Hou and co-workers in 2004, thanks to the use of group 3 “post-metallocene” catalysts [27].

Complex **2**, when treated with 1 equiv. of [Ph_3_C]^+^[B(C_6_F_5_)_4_]^−^ afforded a multiblock sPS-*co*-PE copolymer with styrene incorporation content up to 87 mol %. Unlike other reports concerning styrene-ethylene copolymerization, neither PE nor PS homopolymer side-production was observed. Furthermore, those systems featured remarkable productivities (790–2300 kg·mol^−1^·h^−1^) even when operating under mild conditions (2 min, *T*_polym_ = 25 °C) (it should be noted that this is the starting polymerization temperature; because of the large exothermicity of styrene polymerization, the reaction temperature rises up to ca. 100–150 °C within a few seconds), affording multiblock copolymers, even at low amount of incorporated ethylene. QM/MM studies on first insertion of styrene and ethylene were carried out, suggesting that both insertions are competitive [28]. Moreover, it was demonstrated that 2,1-insertion via a *re*-coordination pattern is more thermodynamically favorable if the first monomer inserted is a styrene. Second insertion of styrene or ethylene were also calculated, showing that insertion of a styrene into an ethylene pre-inserted species is more thermodynamically preferable than insertion of ethylene, but also that ethylene insertion into a styrene pre-inserted is kinetically and thermodynamically favored. This alternating priority between both monomers is in favor of the production of a copolymer. Furthermore, if a second styrene monomer is inserted into a styrene pre-inserted species, it prefers to be coordinated with its *si* face, because of the chirality of the active species as well as repulsive interaction between the methyl groups of the cyclopentadienyl moiety and the phenyl of the incoming styrene. Ligand bulkiness appears to be the predominant factor for syndioselectivity of such a catalyst [28].

Independently, our group described the same year allyl *ansa*-lantanidocene catalysts **9** for syndioselective styrene polymerization [29], and further studies demonstrated their ability to produce sPS-*co*-PE materials [30]. Contrasting with other catalytic systems used for styrene-ethylene copolymerization, those catalysts do not require any co-activator because they involve neutral active species. Higher styrene contents (up to 97 mol %) were obtained than with the half-sandwich scandium complex **2** developed by Hou. Despite the harsher polymerization conditions required (for instance, a temperature above 60 °C,), productivity values were generally one order of magnitude lower compared to the latter catalytic system. The microstructure of the copolymer formed corresponds to isolated ethylene units or very short ethylene sequences randomly distributed over the syndiotactic polystyrene chains, even at quite high incorporated ethylene rates; this is another major distinctive feature as compared to the abovementioned Hou system. Concerning the mechanism of styrene polymerization catalyzed by **9**, it was suggested that the first step is dissociation of the THF molecule, followed by monomer coordination-insertion into the allyl group. Indeed, chain-ends of oligomers were analyzed and were found to be systematically capped by allyl-end groups. Further detailed ^13^C-NMR studies of these oligomers revealed both initiation and propagation steps also proceed via 2,1-insertions [31]. The propagation process is under chain-end stereocontrol, as revealed by the relative intensities of the four heptads *rrrrr*, *mrrrrr*, *rmrrrr* and *rrrmrrr* that match well with Bernoullian statistics. Additional DFT calculations on the four first styrene insertions demonstrated that formation of sPS is thermodynamically controlled [32].

The first ESI materials containing syndiotactic PS sequences obtained with a group 4 metal catalyst was described by Park and co-workers [33]. In combination with 2000 equiv. of MMAO, complex **20** led to the formation of sPS-PE materials featuring a high styrene content (64–80 mol %). However, the exact level of syndiotacticity was not quantified. The origin of this selectivity was not clearly established but the simultaneous presence of both oxo- and pinacolo-bridges in the ligand is suspected by the authors to be part of the answer. Later on, Naga et al. reported styrene-ethylene copolymerizations performed using trivalent Ti(acac)_3_ **18** in combination with TIBAL and MMAO [34]. The syndiotacticity level was also not quantified and activities of the catalytic system were quite low. Genuine sPS-PE copolymer was found in the soluble fractions obtained from extraction of the crude material, featuring a block-like microstructure containing long sPS segments separated by ethylene-ethylene sequence. However, the real microstructure of this copolymer remains quite unclear.

Hou and co-workers reported the synthesis of a new series of half-sandwich fluorenyl scandium dialkyl complexes **6a**–**6b**–**6e**–**6h** [35]. When activated with 1 equiv. of [Ph_3_C]^+^[B(C_6_F_5_)_4_]^−^, in combination with 15 equiv. of Al*^i^*Bu_3_, those ternary catalytic systems were found to be highly active in syndioselective styrene homopolymerization and styrene-ethylene copolymerization, exhibiting productivities comparable with those obtained using the cyclopentadienyl-scandium dialkyl complex **2**. ^1^H- and ^13^C-NMR spectrometry analyses of the copolymers revealed an incorporation of styrene ranging from 38 to 80 mol % and syndiotactic ratios above 90% (likely estimated at the diad level). DFT studies were carried out in order to better understand why the presence of Al*^i^*Bu_3_ significantly accelerates the polymerization process. They demonstrated that Al*i*Bu_3_ can capture the coordinated THF molecule from the precursor to give a THF-free cationic active species after activation by [Ph_3_C]^+^[B(C_6_F_5_)_4_]^−^. Calculated energy barriers for the first styrene insertion showed that this initiation step is much faster if the active species is THF-free, because of the steric repulsion from THF. This steric repulsion becomes more and more important with the increasing polystyrene chain length. Furthermore, they calculated *R* and *S* configurations for the corresponding products of the coordination and insertion of the second and third styrene molecules and highlighted that lower barriers are obtained for opposite configuration of the previous inserted monomer, hence resulting in syndiotactic polystyrene via a chain-end stereocontrol.

Only very few publications have addressed isotactic styrene-ethylene polymerization. The **21a**/MAO binary catalytic system was reported to be efficient for the production of isotactic polystyrene containing isolated ethylene units [36]. Depending on the ethylene content (from 0.5 to 50 mol %), the microstructure varied from isotactic polystyrene blocks linked by an ethylene unit to an alternating styrene-ethylene copolymer. The ^13^C-NMR spectra revealed no signal relative to ethylene units inserted between two iPS blocks with opposite relative configuration, indicating that propagation stereocontrol is under an enantiomorphic site control.

### 2.2. Propylene

sPS-*b*-aPP block copolymers have been synthesized by sequential polymerization of propylene catalyzed by **14c**/MAO followed by addition of styrene and TIBAL [37]. The proportion of propylene in the final material can be controlled by polymerization times. However, this process also provides atactic polypropylene (aPP) and sPS homopolymers as mixtures with the copolymer, which can be isolated by successive (tedious) extractions with different solvents. The thermal behaviour of the isolated copolymer revealed two glass-transition temperatures and one melting point, consistent with the thermal behaviour of both sPS (one *T*_m_ and one *T*_g_) and aPP (only one *T*_g_), which confirms the diblock structure. This copolymer can be used as an effective compatibilizer for sPS-iPP blends [38]. Very recently, Li and co-workers described the synthesis of styrene-propylene copolymer with an ajustable styrene content and a controlled microstructure [39]. Using the well-known binary system **2**/[Ph_3_C]^+^[B(C_6_F_5_)_4_]^−^, they isolated multi-block copolymers containing sPS blocks, aPP blocks and styrene-propylene sequences. Styrene incorporation (10–85 mol %) can be controlled by the styrene-to-propylene feed ratio.

Concerning isoselective styrene-propylene copolymerization, diblock iPS-*b*-iPP was obtained by sequential polymerization of the two monomers using a Ziegler-Natta catalyst (undefined); when added to iPS-iPP blends, this diblock copolymer was found to significantly improve the mechanical properties of such blends [40]. Proto and co-workers also reported that complex **21a** activated by MAO can produce a stereoregular styrene-propylene multiblock copolymer composed of short iPP blocks between long iPS blocks [41]. Full assignment of ^13^C-NMR resonances revealed that the two monomers copolymerize via opposite regioselectivity, resulting in tail-to-tail and head-to-head sequences between two homopolymers blocks. The styrene content in the resulting material was found above 60 mol %, contrasting with other reported styrene-propylene copolymers.

## 3. Copolymerization with Dienes

### 3.1. Butadiene and Isoprene

**Figure 4:**
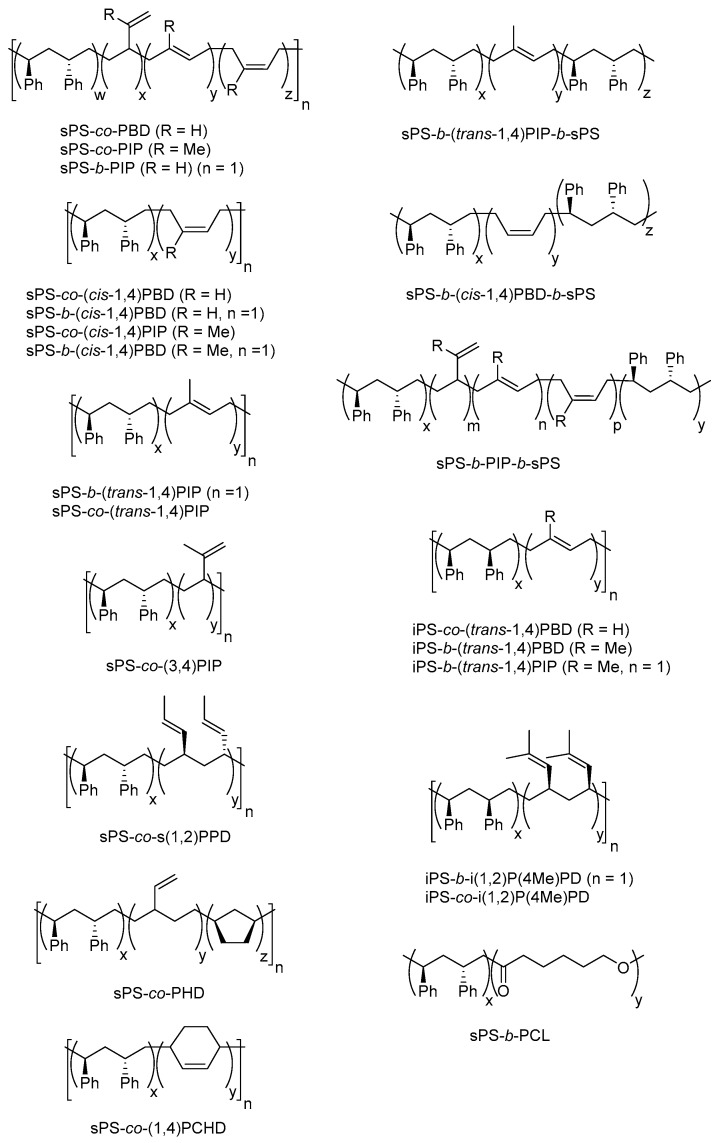
Structure of stereoregular styrene-diene and styrene-caprolactone copolymers.

Stereospecific copolymerization of styrene with butadiene and isoprene has been widely studied since 25 years. In a first approach, polymerizations were attempted in the presence of titanium-based catalysts, well-known to be efficient toward styrene syndiospecific polymerization. After the advent of the “post-metallocenes” rare-earth catalysts, this new class of complexes was explored towards isoprene- and butadiene-styrene copolymerizations, giving access to new copolymers with better controlled microstructures. Copolymer structures and properties are shown in Figure 4 and Table 3.

#### 3.1.1. Group 4 Metallocene Catalysts

One of the first syndiotactic styrene-isoprene copolymers was disclosed by Zambelli and co-workers [42]. In the presence of **12a**/MAO, styrene and isoprene copolymerized to give a random sPS-*co*-PIP copolymer. However, the productivity was quite low and the syndiotacticity was not quantified. In 2000, Bowen and co-workers reported the full characterization of styrene-butadiene copolymers produced by a series of Ti(III) and Ti(IV) catalysts (**12a**–**12b**–**14a**–**15a**–**18**), without contamination by homopolymers [43].

In combination with MAO, CpTiCl_3_ (**12a**) was found to be the most active catalytic system among the series, featuring moderate productivities and narrow dispersity values below 2. Butadiene units were mostly found as 1,4 units (7–15 mol % of 1,2 insertion). Later on, these authors managed to increase the range of styrene incorporation (15–92 mol %) using the same catalytic system [44]. The effect of the substitution of the cyclopentadienyl moiety of half-sandwich titanium(IV) catalysts regarding styrene-butadiene and styrene-isoprene copolymerizations was also studied [45]. By comparison of the copolymer microstructures obtained using complexes **12a**–**13**–**14b**/MAO, it was found that the highest BD content was obtained with complex **14b** whereas the highest amount of IP content was obtained with catalyst **12a**. Surprisingly, the resulting styrene-butadiene materials featured a blocky structure while styrene-isoprene polymers had a random distribution. Moreover, the ratio of BD 1,4-insertion is not affected by the catalyst whereas the IP 1,4-insertion is favored by catalysts **12a** and **13**. Styrene-butadiene block copolymers were also synthesized by living butadiene polymerization catalyzed by the **12a**/MMAO binary system, followed by sequential addition of styrene, affording a sPS-*b*-PBD copolymer with a highly syndiotactic sPS block ([*r*]^4^ > 95%) and a PBD block with *cis*-1,4 units (>77%) [46]. When changing the catalytic system for **14a**/[Ph_3_C]^+^[B(C_6_F_5_)_4_]^−^/Al(oct)_3_, a better control of the livingness of the copolymerization and hence of the block lengths was achieved [47]. Further on, triblock sPS-*b*-(*cis*-1,4)-PBD-*b*-sPS was synthesized following the same procedure [48]. Unlike non-regular SBS polymers synthesized via anionic processes, this new polymer has a high melting point above 272 °C and exhibits higher chemical and heat deformation properties. Styrene-butadiene and styrene-isoprene syndiotactic copolymers were also prepared using titanium precursors bearing oxo- ligands. Complexes **16** and **19**, activated with MAO, afforded materials with a high *cis*-1,4 regioregularity for both BD and IP, featuring a blocky microstructure in the first case and a pseudo-random one in the last case [49,50]. Interestingly, Ti(IV) complex **17** was not able to produce styrene-butadiene copolymer but only a mixture of homopolymers. These results highlighted the important role of the oxo-ligand in styrene-diene copolymerization process.

Isospecific styrene-butadiene copolymerization using titanocene catalyst was first achieved by Proto et al. On the one hand, using complex **21a** activated by MAO, they obtained a random iPS-*co*-PBD copolymer in which BD is predominantly *trans*-1,4 inserted [51]. One the other hand, diblock iPS-*b*-PBD was synthesized by sequential polymerization catalyzed by complex **21b**/MAO, starting from styrene followed by butadiene [52]. It was demonstrated that the cumyl group in complex **21b** afforded a better livingness of both styrene and butadiene homopolymerizations compared to complex **21a** bearing *tert*-butyl groups, making it a better candidate for the synthesis of diblock polymers. In both cases, the incorporated styrene contents covered a wide range (15–97 mol %). However, molecular weights are higher and dispersity values are significantly narrower in the diblock material than in the random one, thanks to the living character of polymerization process used to obtain the diblock material. Interestingly, BD insertion is mainly *trans*-1,4 specific in both copolymers, contrasting with the other group 4 catalysts described in this part which copolymerize styrene in a syndiospecific manner and BD in a *cis*-1,4 specific manner. Same iPS-*b*-(1,4)PBD with prevalently *trans*-1,4 structure was also obtained using zirconium complex **22** bearing a similar OSSO-type ligand [53]. This catalyst was found to have a better thermal robustness than its titanium analogue. On the other hand, *rac*-bis-indenyl zirconocene catalysts **23a**–**23b** were tested in the styrene-butadiene copolymerization and compared with their titanocene analogue **23c [54]**. All of them produced copolymers containing isotactic PS sequences and *trans*-1,4 PBD units, and the amount of *trans*-1,4 units decreased with decreasing the styrene content. However, *cis*-1,4 insertion became predominant in copolymers produced by **23c** and containing less than 30 mol % of styrene, whereas *trans*-1,4 insertions were always predominant in copolymers produced by zirconocenes catalysts, whatever the BD molar fraction was.

#### 3.1.2. Group 3 Catalysts

For 15 years, efforts were focused on the development of “post-metallocenes” catalysts based on group 3 complexes. When titanocenes catalysts were more widely studied for styrene-butadiene copolymerization, the catalytic systems based on rare-earth complexes were found to be particularly efficient for the styrene-isoprene copolymerization, constituting the majority of the reported works.

Half-sandwich scandium complex **2** was reported by Hou and co-workers as an efficient catalyst for styrene-isoprene copolymerization [55]. Diblock, triblock and multiblock sPS-PIP were obtained by sequential or random copolymerizations, respectively. In all the cases, the styrene content can be adjusted by changing the monomers feed ratio. However, no regiospecificity regarding isoprene was observed, but a mixture of 1,4 and 3,4 insertions. The first sPS-*b*-(*trans*-1,4)PIP diblock copolymer was synthesized by Visseaux and co-workers [56]. Inspired by the half-sandwich structure of the titanocenes complexes presented in the previous part, they developed new catalytic systems based on half-lanthanidocenes complexes for styrene-isoprene copolymerization. Associated with *n*-butylethylmagnesium as co-activator/chain-transfer agent, the borohydrido neodymium complex **7** afforded a copolymer constituted of a short sPS block ([*r*]^6^ = 85%) connected to a 98% of (*trans*-1,4)PIP block.

The development of a new family of neutral complexes bearing allyl ligands gave access to highly productive catalytic systems for styrene-isoprene copolymerization. Allyl *ansa*-neodymocene complex **9**, first described by our group for syndiospecific styrene polymerization, also proved effective for the production of random sPS-*co*-(*trans*-1,4)PIP copolymers [57]. The productivity was quite high (up to 1025 kg·mol^−1^·h^−1^ at 80 °C) and the styrene content (70–94 mol %) can be controlled by the initial monomer feed ratio. On the other hand, multiblock iPS-*co*-(*trans*-1,4)PIP copolymer as well as diblock iPS-*b*-(*trans*-1,4)PIP were synthesized respectively by simultaneous or sequential monomer copolymerization catalyzed by the isospecific *ansa*-bis(indenyl) yttrium complex **10** [58]. DFT studies of the isoselective styrene homopolymerization suggested that the initiation step is insertion of styrene into the Ln-C(allyl) bond and that both initiation and propagation proceed via 2,1 insertions. These results are similar to those obtained in the case of syndioselective styrene polymerization catalyzed by complex **9** [59]. However, an enantiomorphic site control seemed to be here at the origin of the isospecific behavior of the catalyst.

Interestingly, none of the catalysts previously described was able to produce styrene-isoprene copolymers containing *cis*-1,4 or 3,4 units. This limitation was overcome by the use of a chain-shuttling polymerization process involving complex **2**, known to be very efficient for syndioselective styrene polymerization, and complexes **1** or **11**, as regioselective catalysts for *cis*-1,4 or 3,4 isoprene polymerization [60]. Activated by [Ph_3_C]^+^[B(C_6_F_5_)_4_]^−^ and in the presence of Al*^i^*Bu_3_ as the chain-shuttling agent, complex combinations **2** + **1** or **2** + **11** produced multiblock sPS-*co*-(*cis*-1,4)PIP or sPS-*co*-(3,4)PIP copolymers, respectively. This protocol gave access for the first time to highly stereo- and regioregular styrene-butadiene or isoprene copolymers ([*r*]^4^ > 99%, *cis*-1,4 > 97% and 3,4 ca. 90%). Independently, the synthesis of sPS-*co*-(*cis*-1,4)PIP materials was also described by Cui and co-workers using lutetium-allyl complex **8** activated by [Ph_3_C]^+^[B(C_6_F_5_)_4_]^−^ [61]. Diblock and multiblock copolymers with a broad range of styrene content (6–83 mol %) and *cis*-1,4 isoprene insertion up to 70% were obtained by sequential or competitive styrene-isoprene copolymerization, respectively. The same catalytic system was previously tested in styrene-butadiene copolymerization [62]. Interestingly, only a diblock sPS-*b*-(*cis*-1,4)PBD copolymer was obtained when styrene and butadiene were mixed in the presence of **8**/[Ph_3_C]^+^[B(C_6_F_5_)_4_]^−^, as judged by the absence of ^13^C-NMR spectroscopy resonance from the carbon-carbon linkage between the two monomers. A possible explanation was that butadiene polymerizes faster than styrene but is not easily inserted as soon as styrene starts to polymerize. As a result, when sequential polymerization was run, the expected diblock copolymer was recovered only when styrene was added after butadiene. In the reverse case, only a mixture of homopolymers was obtained. The same observation was made when using **2**/[Ph_3_C]^+^[B(C_6_F_5_)_4_]^−^. Furthermore, the synthesis of stereoregular sPS-*b*-(*cis*-1,4)PBD star-shaped copolymer was achieved by addition of *para*-divinylbenzene on the living polymer chain and subsequent cross-linking of the resulting terpolymer [63].

Very recently, syndioselective copolymerization of styrene with butadiene was performed by Cui and co-workers using catalysts based on several group 3 metals (Nd, Y, Tm and Sc) [64]. Depending on the metal center (and so the ionic radius), the copolymer microstructure varied from diblock (with Nd complex **5a**) to tapered (Y complex **5b**), gradient (Tm complex **5c**) and finally random (Sc complex **5d**). Those different materials could be obtained thanks to the difference of reactivity between butadiene and styrene. Indeed, butadiene is much more reactive than styrene when the neodymium complex is involved, and that discrepancy is lesser and lesser pronounced with decrease of ionic radius. Hence, with the scandium catalyst, both monomers have similar reactivity. DFT studies revealed that, with the neodymium complex, insertion of butadiene is thermodynamically favored whatever the last inserted unit is (styrene or butadiene), producing a diblock copolymer. On the other hand, for the scandium complex, intermediates resulting from self-propagation and cross-propagation have the same energy, producing a random copolymer. However, the origin of such a discrepancy was still unclear. Knowing that insertion of butadiene is sterically more demanding than that of styrene, the authors suggested that the larger the metal center is, the less crowded the coordination sphere is and thus the easier the insertion of butadiene should be. Moreover, the PS segments were highly syndiotactic in all the copolymers while regio/stereochemistries of the PBD segments were different depending on the nature of the metal center (10%–12% of *trans*-1,4 insertion whatever the metal of the complex, and from 73% to 52% of *cis*-1,4 and from 15% to 38% of 1,2 insertion when going from neodymium to scandium complex). Mechanical tests were carried on the 4 copolymers with different microstructures. Diblock material featured moderate tensile strength and elongation at break (100%). In tapered and gradient materials, butadiene-styrene sequences are present in minor quantity but enough to compatibilize the sPS and PBD phases of the copolymer, imparting to the polymer the mechanical properties of both, and especially a high extensibility (200%). The random material is composed of a unique and homogeneous phase that behaves like a brittle material (elongation at failure = 17%). However, this value is higher than that of pure sPS, due to a lower crystallinity of the copolymer induced by the presence of butadiene.

### 3.2. Other Conjugated and Non-Conjugated Dienes

Styrene copolymerization with higher or bulkier dienes has also been reported (see Table 4 and Figure 3). For instance, Longo et al. described the syndiospecific copolymerization of styrene with 1,3-pentadiene (PD) promoted by **12a**/MAO catalytic system [65]. When the polymerization was performed at −20 °C, blocky sPS-*co*-s(1,2)PPD was obtained, with a majority of syndiotactic 1,2 sequences of pentadiene. However, when the polymerization was run at 20 °C, *cis*-1,4 sequences were also observed. Styrene-1,3-pentadiene copolymerization was also probed in the presence of **21a** and **21b** activated by MAO [66]. In this case, random copolymers containing a majority of 1,2 pentadiene units were recovered. The tacticity of the PS sequences was not quantified, probably due to the fact that both complexes mostly afforded short PS homosequences. Using the same catalyst **21b**, styrene and 4-methyl-1,3-pentadiene ((4Me)PD) were efficiently copolymerized [67,68]. Both diblock iPS-*b*-i(1,2)P(4Me)PD and nearly alternated iPS-*co*-i(1,2)P(4Me)PD copolymers were obtained.

Rare-earth complexes were also used in the synthesis of new families of stereoregular PS-based copolymers. In combination with [Ph_3_C]^+^[B(C_6_F_5_)_4_]^−^, complexes **4a**–**4c** led to an original styrene-*co*-1,5-hexadiene material containing sPS sequences and two different polyhexadiene units: methylene-1,3-cyclopentane (MCP) and vinyltetramethylene (VTM) [69]. Interestingly, at fixed St/HD feed ratio, the sPS content increased with the bulkiness of the cyclopentadienyl moiety of the complex used. Detailed NMR spectroscopy analysis confirmed the *trans*-configuration of the MCP units and the absence of St-MCP sequences, presumably due to the steric hindrance between the two units.

Regio- and stereoregular styrene-1,3-cyclohexadiene (CHD) copolymers were prepared in the presence of a series of group 3 complexes (**6a**–**6j**) [70]. Under the same conditions their activities were found in the order **6e** > **6j** > **6b** > **6a** > **6i** > **6g** > **6f** = **6d** > **6h** > **6c**. No clear trend concerning the nature of the ligand or the nature of metal center was established, i.e., the best ligand in terms of activity depends on the nature of metal center and *vice-versa*. This is actually a question of good balance between the bulkiness of the ligand and the size of the metal center. Using **6e**/[Ph_3_C]^+^[B(C_6_F_5_)_4_]^−^, a range of random sPS-*co*-(1,4-PCHD) copolymers was obtained, containing 26–88 mol % of styrene.

### 3.3. Other Comonomers

Reports on copolymerization of styrene with a comonomer that is not an olefin or a conjugated diene are very rare. To our knowledge, there is only one example reporting sequential syndiotactic polymerization of styrene followed by polymerization of ε-caprolactone catalyzed by complexes **2** and **3** [71]. Preliminary stress-strain studies were carried on the resulting sPS-*b*-PCL and revealed better tensile strength and flexibility than the corresponding homopolymers or blends, demonstrating the potential of this new material.

## 4. Copolymerization with Styrene Derivatives

### 4.1. Styrene with Substituted Styrene Derivatives

#### 4.1.1. Alkyl-Substituted Styrene Monomers

After the disclosure of sPS, polymerization of styrene derivatives by syndiospecific titanium-based catalysts was attempted. Thus, CpTiCl_3_ (**12a**)/MAO and Ti(O*t*Bu)_4_ (**15b**)/MAO were found to be efficient catalytic systems for the production of syndiotactic polyalkylstyrene or halogenated polystyrene [5,72]. Then, copolymerization of styrene with those substituted styrene monomers was deliberately explored. Using Ti(OMen)_4_ (**15c**)/MAO, Ti(Bz)_4_ (**15b**)/MAO, CpTiCl_3_ (**12a**)/Ph_2_Zn/MAO, (*n*BuCp)_2_TiCl_2_/Ph_2_Zn/MAO or (Ind)TiCl_3_ (**13**)/Ph_2_Zn/MAO systems, true copolymers were obtained [72,73,74,75]. However, the microstructure of the materials, in particular syndiotacticity levels, were not systematically investigated. Rabagliati et al. also reported the synthesis of PS-*co*-P(*p*-*t*Bu)S copolymers being syndiotactic in nature whatever their composition; catalyst activities were found in the order Ind_2_ZrCl_2_ < (*n*BuCp)_2_TiCl_2_ < CpTiCl_3_ (**12a**) [76,77]. Later on, PS-*co*-P(2,4-Me_2_)S copolymers were obtained featuring a syndiotacticity decreasing with the (2,4-Me_2_)S content [78]. Using Cp*TiCl_3_ (**14b**) in combination with B(C_6_F_5_)_3_ and Al(Oct)_3_, random living copolymerization of styrene and *p*-methylstyrene led to copolymers with syndiotactic sequences [79,80]. Isotactic diblock iPS-*b*-iP(*p*-Me)S and triblock iP(*p*-Me)S-*b*-iPS-*b*-iP(*p*-Me)S copolymers were also synthesized through living sequential polymerization catalyzed by **21b**/MAO [67].

#### 4.1.2. Polar Styrene Monomers

Copolymerization of styrene with polar styrene monomers is very challenging, due to the significantly different reactivity of these two monomers, resulting in low polar monomer incorporation, losses of activity and stereoselectivity, and low molecular weights. Consequently, it appeared to be much easier to obtain diblock copolymers rather than random or multiblock copolymers. Murata and co-workers described the synthesis of syndiotactic diblock sP(*p*-Me)S-*b*-s(*p*-HO)S material obtained via sequential block copolymerization of *p*-methylstyrene and 4-(*tert*-butyldimethylsilyloxy)styrene (TBDMSS) catalyzed by **14a**/B(C_6_F_5_)_3_/Al(Oct)_3_ and subsequent desilylation of the resulting P(TBDMSS) block [81]. The synthesis of diblock sPS-*b*-sP(TBDMS)S using (Ind)TiCl_3_ (**13**)/MAO was also described; however, no deprotection of the silyl group was performed on this copolymer [82].

**Figure 5:**
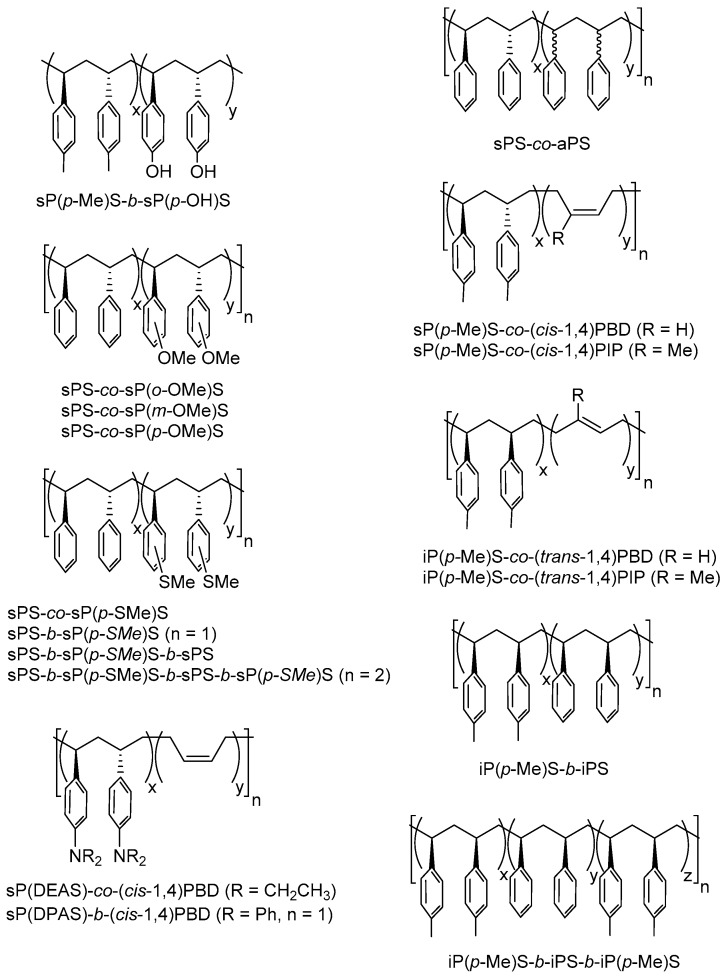
Structure of stereoregular styrene derivative copolymers.

Thanks to the development of highly syndiospecific rare-earth catalysts (*vide supra*), a new family of copolymers became accessible. Recently, Hou and co-workers reported the homo- and copolymerization of methylthiostyrene (MTS) with styrene catalyzed by **5b**/[Ph_3_C]^+^[B(C_6_F_5_)_4_]^−^/TIBAL [83]. The resulting copolymer is highly syndiotactic and the MTS content ranges from 11 to 93 mol %, in good agreement with the initial feed, because the monomer reactivity ratio is close to 1. Di-, tri- and tetrablocks materials were also successfully synthesized, demonstrating the quasi-livingness of the catalytic system. Later on, this group reported the copolymerization of styrene with methoxystyrene (MOS) catalyzed by the same yttrium catalyst (**5b**) than in the case of methylthiostyrene [84]. sPS-*co*-sP(OMe)S copolymers can be obtained as well featuring syndiotactic sequences whatever the position of the methoxy group is (*ortho*-, *meta*- or *para*-). However, the macromolecular topology was different depending on the MOS monomer involved: tapered with *o*MOS, gradient with *m*MOS and an almost random with *p*MOS. This is due to the different reactivity ratio of MOS monomers and styrene which is more pronounced in the case of *o*MOS than in the case of *m*MOS, while *p*MOS and styrene appeared to have similar reactivity ratios. Energies of insertion of either MOS or styrene unit after the insertion of MOS were calculated by DFT. For *o*MOS monomer, the energy difference for the two routes is in favor of *o*MOS insertion, resulting in a tapered copolymer. For *p*MOS monomer, the activation energies for both routes are rather similar, explaining why a random copolymer is preferably formed. Those differences come from the different insertion mode of *o*MOS and *p*MOS, resulting from the various position of the methoxy substituent. Furthermore, only P(*o*OMe)S homopolymer was obtained when the copolymerization of *o*OMeS and styrene was catalyzed by **2** while a true copolymer was isolated when using catalyst **5b**. This result can be explained by the fact that the coordinated pyridine moiety of the ligand in the catalyst **5b** reduces the Lewis acidity of the metal center, weakening the interaction between the methoxy group of the incoming monomer and thus facilitating the styrene coordination-insertion. Polar styrene copolymer structures and properties are shown in Figure 5 and Table 5.

### 4.2. Substituted Styrene Monomers with Other Monomers

Several works have addressed the copolymerization of substituted styrene derivatives with other monomers, like isoprene or butadiene, involving catalytic systems already described for stereoregular styrene copolymerization. Copolymers structures are depicted in Figure 5. Proto and co-workers studied the copolymerization of *p*-methylstyrene and reported the synthesis of isotactic iP(*p*-Me)S-*b*-(*trans*-1,4)PBD copolymer by sequential copolymerization promoted by **21b**/MAO [67]. Using the *t*Bu-substituted analogue, complex **21a**, isotactic copolymers iP(*p*-Me)S-*co*-(*trans*-1,4)PBD and iP(*p*-Me)S-*b*-(*trans*-1,4)PIP were obtained [85]. In the presence of **19** activated by MAO, (*p*-Me)S copolymerized with butadiene and isoprene in a syndiospecific manner, affording the corresponding sP(*p*-Me)S-*co*-(*cis*-1,4)PBD and sP(*p*-Me)S-*b*-(*cis*-1,4)PIP copolymers. These new materials were synthesized for a wide range of compositions (χ_(*p*Me)S_ = 0.2–0.9). Consistent with the results obtained for copolymerization of non-substituted styrene with isoprene and butadiene, catalyst **19** was found to be syndioselective towards (*p*-Me)S and *cis*-1,4 selective towards isoprene and butadiene, whereas catalyst **21a** was found to be isoselective towards (*p*-Me)S and *trans*-1,4 selective towards isoprene and butadiene [49,51]. In addition, the copolymers featured blocky or random microstructures depending on whether they were produced with catalyst **19** or **21a**, respectively.

Syndioselective copolymerizations of butadiene with amino-functionalized styrene derivatives, namely *p*-*N,N*-dimethylaminostyrene (DMAS), *p*-*N,N*-diethylaminostyrene (DEAS) and *p*-*N,N*-diphenylaminostyrene (DPAS), were performed in the presence of the **4c**/[Ph_3_C]^+^[B(C_6_F_5_)_4_]^−^ system [86,87]. Multiblock sP(DEAS)-*co*-(*cis*-1,4)PBD and diblock sP(DPAS)-*b*-(*cis*-1,4)PBD copolymers were successfully synthesized, both featuring syndiotactic amino-functionalized PS sequences and high *cis*-1,4 butadiene insertion content (ca. 85%). In contrast, the copolymerization of DMAS with butadiene led to the formation of materials exhibiting bimodal distributions and average molecular mass values decreasing when DMAS content is increased. The authors suggested that a termination reaction could occur by proton transfer from the incoming DMAS monomer and the growing chain, generating a “dead” copolymer chain and a new active species. This is possible because the amino group of DMAS can be coordinated to the metal center and facilitates C–H activation in alpha, followed by transfer of this hydrogen to the polymer chain. On the contrary, for DEAS and DPAS, coordination of bulkier amino groups was disfavored, thus disabling the termination process previously mentioned.

### 4.3. Stereoblock Polystyrene

There are numerous example of copolymers containing both sPS and aPS segments at the same time. However, they are principally represented by graft or diblock copolymers, synthezised through multi-step processes (see Section 6 for details); there are very few examples of stereoblocks PS copolymers obtained through a one-pot procedure involving a single catalytic system. The most relevant example is a multiblock sPS-*co*-aPS synthesized by Longo et al. through an elegant process involving the hapto-flexible catalyst **17** [88]. Depending on the temperature, the ether group of the ligand is coordinated to the titanium or not, affording different active species: a syndiospecific one in which the ether function is claimed not to be coordinated (around 19 °C), and an aspecific one with the coordinated ether (0–5 °C). As a result, if the polymerization is carried out at a medium temperature (10–15 °C) both species would coexist and a stereoblock copolymer is thought to be formed, as judged by the monomodal and narrow molecular weight distribution observed by GPC analysis.

## 5. Terpolymerization

**Figure 6:**
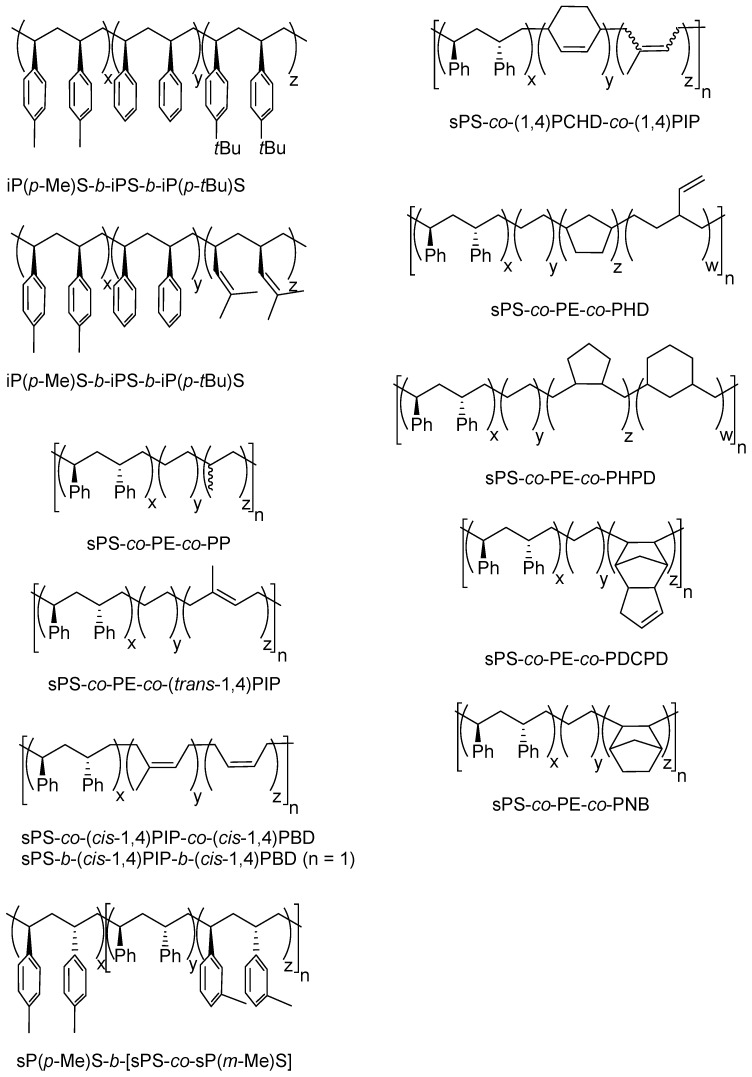
Structure of stereoregular styrene-based terpolymers.

Incorporating a third monomer in a styrene copolymer chain can give access to new materials with unusual and unprecedent properties. Furthermore, when the copolymerization of styrene with another comonomer (especially a bulky one) fails due to a steric congestion, addition of a smaller ethylene can help to “re-activate” active site of a catalyst. There are very few examples of such terpolymerization reactions catalyzed by titanium-based catalyst. Resulting terpolymer structures and properties are sumarized in Figure 6 and Table 6.

Living polymerization of (*p*-Me)S with Cp*TiCl_3_ (**14b**)/B(C_6_F_5_)_3_/Al(Oct)_3_ followed by sequential addition of a S/(*m*-Me)S mixture afforded the syndiotactic terpolymer sP(*p*-Me)S-*b*-[sPS-*co*-sP(*m*-Me)S]. After partial chlorination of the methyl groups, this material was induced in reaction with a living poly(2-vinylpyridine)-Li polymer chain to give a new block grafted syndiotactic terpolymer [80]. In the presence of **21b**, isotactic triblock copolymers composed of polystyrene, poly(*p*-Me)styrene and poly(*p*-*t*Bu)styrene or poly(4-Me)pentadiene blocks can be obtained via sequential polymerization [67].

Thanks to the development of rare-earth complexes, significant breakthroughs in the field of stereoselective styrene terpolymerization were achieved. The synthesis of styrene-ethylene-propylene terpolymer containing large amounts of styrene (5–32 mol %) was achieved by Li and co-workers [39]. Scandium based system **2**/[Ph_3_C]^+^[B(C_6_F_5_)_4_]^−^ efficiently produced random sPS-*co*-PE-*co*-PP polymers featuring highly syndiotactic PS sequences. The ^13^C-NMR resonances from ethylene-styrene and ethylene-propylene sequences were found much more intense than the resonance from the styrene-propylene sequences, probably due to steric reasons and conflicting insertion regiochemistries of styrene and propylene.

Stereoselective terpolymerization of styrene with conjugated dienes was also explored. For instance, the *ansa*-neodymocene **9** was found to be active in styrene-ethylene-isoprene terpolymerization [57]. Same syndio- and regioselectivities as in styrene-isoprene copolymerization were observed, i.e., syndioselectivity towards styrene and (*trans*-1,4) selectivity towards isoprene. Unusually large amounts of styrene were incorporated (41–96 mol %) and the overall composition could be controlled by the initial monomers feed.

Terpolymerization of styrene with butadiene and isoprene was independantly disclosed by Cui et al. and by Hou et al. The first group reported sequential or simultaneous polymerization of those three monomers catalyzed by **8**/[Ph_3_C]^+^[B(C_6_F_5_)_4_]^−^ to give triblock or multiblock terpolymers [61]. The second group also obtained multiblocks polymers using a shuttling catalytic system involving two scandium complexes (**2**, highly active and syndiospecific towards styrene polymerization and **1**, highly active and *cis*-1,4 selective towards butadiene and isoprene polymerization) and TIBAL as the chain-transfer agent (“shuttle”). In all cases, the PS blocks were syndiotactic and the PBD blocks mainly contained *cis*-1,4 units (>96 mol %). However, higher amounts of *cis*-1,4 isoprene units were found in the polymer synthesized via the latter chain-shuttling process than with the one involving the lutetium complex **8**.

Terpolymerization of styrene and isoprene with cyclohexadiene was also performed in the presence of rare-earth complexes **6h**–**6j** [89]. The resulting polymer featured 100% of 1,4-incorporated cyclohexadiene and mostly 1,4 isoprene units (>90 mol %). The nature of the metal center influenced not only the productivity (the smaller the ionic radius, the higher the productivity) but also the polymer microstructure. With the scandium complex **6h** and under the same conditions, a lower styrene content was obtained in contrast with with the two other complexes **6i** and **6j**. Furthermore, mostly (*cis*-1,4) PIP units were observed when Sc complex was involved, whereas mostly (*trans*-1,4) units formed with the lutetium and yttrium congeners.

Efficient terpolymerizations of non-conjugated 1,5-hexadiene (HD) or 1,6-heptadiene (HPD) with styrene and ethylene were achieved by Hou and co-workers using **4c**/[Ph_3_C]^+^[B(C_6_F_5_)_4_]^−^ as catalytic system [69,90]. In the polymer chain, both dienes can be found as two different units: methylene-1,3-cyclopentane (MCP) or vinyltetramethylene (VTM) for 1,5-hexadiene and methylene-1,3-cyclohexane (MCH) or ethylene-1,2-cyclopentane (ECP) for 1,6-heptadiene. While both MCH and ECP sequences were present in a 3/1 ratio in the styrene-ethylene-hexadiene terpolymer, only very small amounts (<1 mol %) of VTM were detected in the styrene-ethylene-heptadiene terpolymer. PS sequences were shown to be syndiotactic in all polymers. Moreover, in the absence of ethylene, styrene-heptadiene copolymerization resulted in the production of a mixture of homopolymers, demonstrating that addition of ethylene can facilitate the production of new materials.

Terpolymerization of styrene and ethylene with cyclic olefins has been scarcely explored [91]. Dicyclopentadiene (DCPD)-styrene-ethylene random terpolymer featuring sPS sequences can be produced in the presence of **2**/[Ph_3_C]^+^[B(C_6_F_5_)_4_]^−^ [92]. As in the case of styrene-heptadiene copolymerization, styrene-DCPD copolymerization cannot take place without addition of ethylene. Accordingly, no styrene-DCPD sequences were detected in the terpolymer. Almost the same result was observed for styrene-ethylene-norbornene terpolymerization [93]. No polymer characterization was provided.

## 6. Stereoregular Diblock and Graft Polystyrene Copolymers

Numerous stereoregular polystyrene-based copolymers reported in the literature, namely diblock and graft copolymers, were not synthesized via regular polymerization processes like those described above, i.e., using processes involving a single catalytic system able to produce the targeted polymer (see Table 7).

For instance, diblock copolymers can be obtained through two-step sequential processes. On the one hand, as soon as the first monomer is polymerized, its chain-ends are chemically modified then. Then, this polymer is used as macroinitiator for the polymerization of the second monomer, involving a second catalytic system. Alternatively, diblock copolymers can also be produced by coupling two homopolymers bearing appropriate chain-ends. Graft copolymers are branched materials made with two types of monomers: one for the main chain and the other one for the pendant chains. These synthetic routes are sometimes a more efficient way to get access to well-defined copolymers containing stereoregular polystyrene sequences due to the difficulty to find a single catalyst effective in polymerization of both styrene and the second monomer targeted. These alternative strategies are described below.

### 6.1. Polystyrene Graft Copolymers

**Scheme 1:**
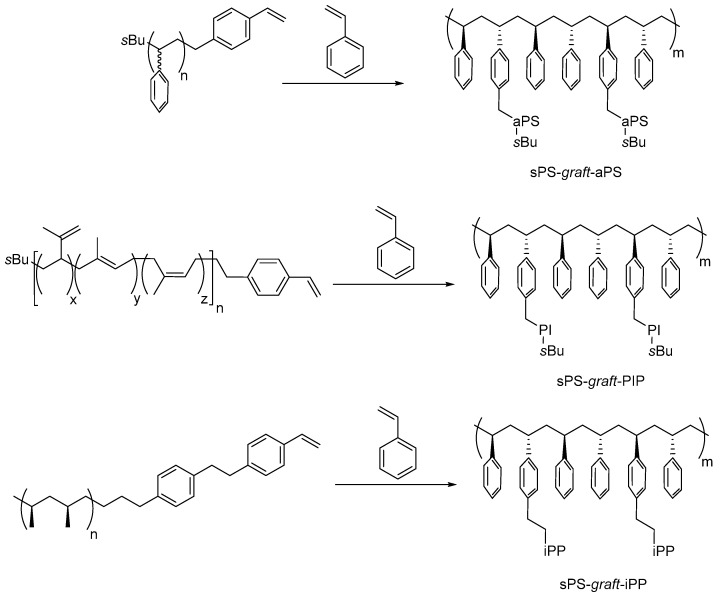
Synthesis and structure of stereoregular graft copolymers obtained via macromonomers.

The first strategy consisted in synthetizing by anionic polymerization a macromonomer having a vinyl chain-end, followed by copolymerization of the latter with styrene in the presence of a syndioselective catalyst (Scheme 1).

This approach was applied by Senoo and co-workers in order to isolate graft copolymers having a sPS main chain and aPS or PIP side chains [94,95]. The styrene-terminated aPS and PIP macromonomers (aPSM and PIPM, respectively) were produced by anionic polymerization, initiated by *sec*-BuLi of the corresponding monomer, followed by nucleophilic substitution between the living polymer chain and *p*-chloromethylstyrene to generate the styrenyl chain-end. Subsequent syndioselective copolymerization was catalyzed by CpTiCl_3_ (**12a**)/MAO. The PIP side-chains microstruture was a mixture of *cis*-1,4 (ca. 70%), *trans*-1,4 (ca. 23%) and 3,4 units (ca. 7%). Further studies demonstrated that, at given mol % of PIPM in the feed, the longer is the PIPM macromonomer, the shorter are the macromolecules and the lower is the number of grafted branches per molecule. In addition, at given PIPM molecular weight, the higher the molar concentration of PIPM in the feed is, the higher the number of grafted branches per molecule is [96].

A similar procedure allowed to synthesize sPS-*graft*-iPP copolymer via propylene polymerization in the presence of the highly isospecific zirconocene **24**/dMAO (dried MAO), 1,2-bis(4-vinylphenyl)ethane and H_2_ to enable formation of iPP-styrenyl macromonomer [97]. As in the case of sPS-*graft*-aPS mentioned above, the macromonomer reactivity and the number of the grafted chains decreased with increasing molecular weight, probably due to the increase in steric hindrance. Furthermore, the number of the grafted chains also decreased with increasing styrene concentrationin the feed, whatever is the molecular weight of the macromonomer, suggesting that the copolymer composition can be controlled by the monomer feed.

Substantial work was achieved on the synthesis of sPS-*graft*-aPS and sPS-*graft*-P(M)MA through Atom Transfer Radical Polymerization (ATRP) of the second monomer onto the sPS main chain. This approach is the opposite to the one described above, since its starts from the syndiospecific polymerization of styrene, followed by chemical modification of the sPS chain to obtain a suitable macroinitiator for ATRP of the second monomer.

**Scheme 2:**
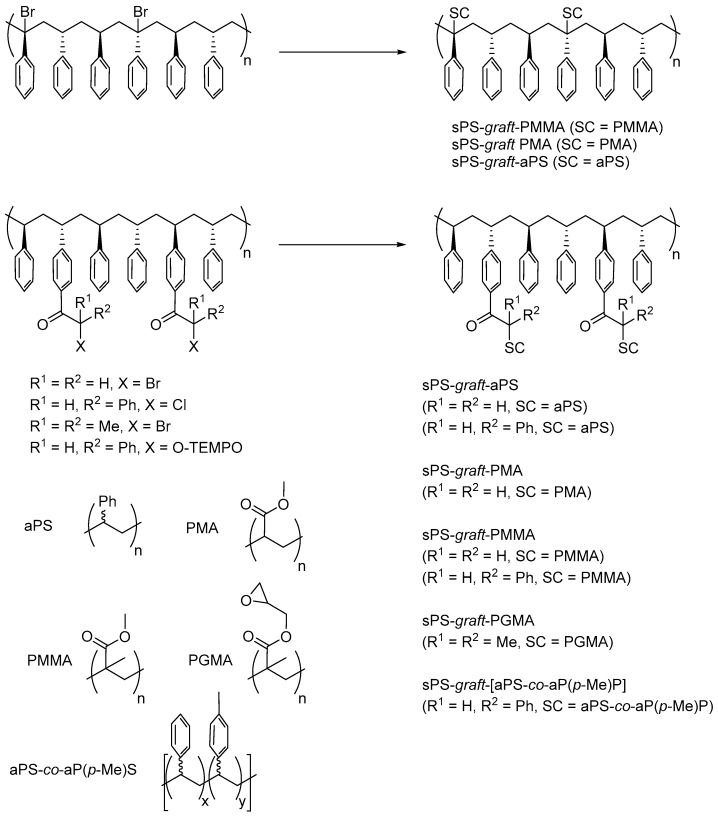
Synthesis and structure of stereoregular graft copolymers obtained via macromonomer.

The advantage of this approach is that ATRP is one of the most powerfull radical processes to synthesize polymers with controlled molecular weight and narrow dispersity [98,99]. For the production of sPS, catalytic systems as CpTiCl_3_ (**12a**) or Cp*Ti(OCH_2_C_6_H_5_)_3_ (**14c**) activated by MAO were used. The modification of the chain was done by bromination onto the olefinic chain or, in most cases, by Friedel-Crafts acetylation to link −C(O)R^1^R^2^X groups on the para-position of the phenyl rings. A range of macroinitiators were thus obtained (Scheme 2), all having C–halogen bonds which efficiently initiate ATRP. By this method were grafted aPS, PMA, PMMA and PGMA side-chains [100,101,102,103]. Further Baeyer-Villiger oxidation of sPS-*graft*-aPS polymers also allowed to transform the ketone into an ester function, the latter could be hydrolyzed to recover both sPS and aPS homopolymers [104]. It was also demonstrated that a sPS-graft-(aPS-*co*-a(*p*-Me)S) terpolymer can be obtained by simultaneous radical copolymerization of styrene and p-methylstyrene initiated by sPS-*graft*-TEMPO macroinitiator [105].

### 6.2. Diblock Copolymers

**Scheme 3:**
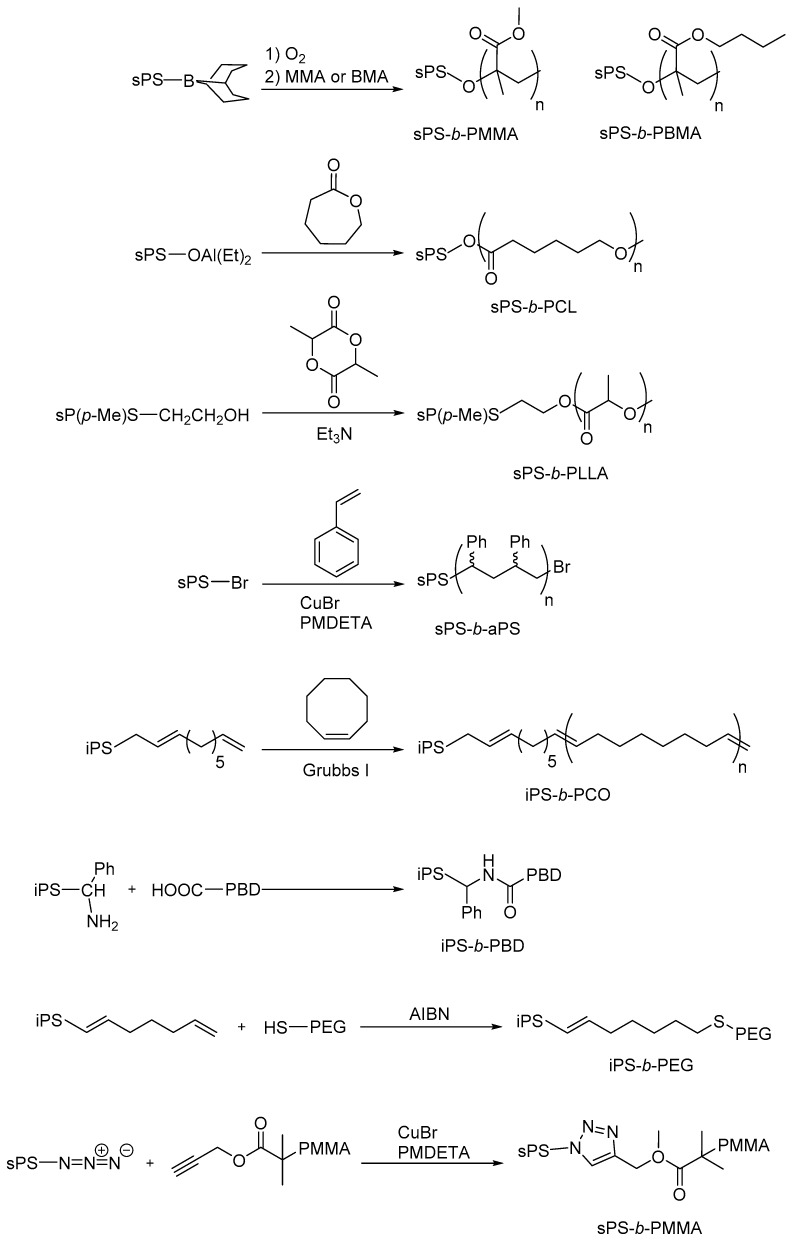
Synthesis and structure of stereoregular diblock copolymers.

Diblock copolymers containing stereoregular PS sequences can be synthesized via sequential polymerization of the two monomers, as described in the first sections of this paper. However, this process involves identifying a suitable catalytic system able to polymerize both monomers with the desired stereo- or regioregularity through a living process. To overcome these limitations, another way consists in polymerizing styrene in the presence of a stereoselective catalyst and a transfer agent to yield stereoregular polystyrene macroinitiators bearing functionalized chain-end, that can also be modified via post-polymerization. Then, the second polymerization initiated by this macroinitiator takes place to provide diblock materials. A range of various sPS-diblock copolymers were obtained using this convenient method (Scheme 3).

The synthesis of sPS-*b*-PMMA and sPS-*b*-PBMA was performed by radical polymerization initiated by the borane-terminated sPS-borabicyclo[3.3.1]-nonane (BBN) [106]. sPS-*b*-PCL and sPS-*b*-PLLA were produced by anionic ring-opening polymerization of ε-caprolactone or L-lactide, respectively [107,108]. sPS-*b*-PCL was found to be effective to improve the compatibilities of polyolefins and PCL blends, and sPS-*b*-PLLA could form nanostructures by self-assembly of the diblock copolymer thanks to the stereoregularity of the PLLA segment. A series of sPS-*b*-aPS copolymers was also synthesized via a two-step procedure involving a sPS-Br macroinitiator prepared by ATRP of styrene [109]. When this copolymer was mixed with sPS-aPS blends, cristallization rates where found in the order sPS > sPS-*b*-aPS ~ blend sPS-*b*-aPS/sPS/aPS > blend sPS/aPS, and a significant improvement of the impact strength of the blends was achieved compared to that of pure sPS and their pure blends [38,110]. More recently, iPS-*b*-PCO copolymer was synthesized via ring-opening metathesis polymerization (ROMP) of cycloctene promoted by Grubbs I catalyst in the presence of iPS-CH_2_CH=CH(CH_2_)_5_CH=CH_2_ macromonomer used as chain-transfer agent [111]. This material seemed also promising as iPS-thermoplastic elastomers blends compatibilizer.

A more trivial way to obtain diblock copolymers with well-defined structure is to generate each homopolymers having proper chain-end to cross-link them in a second time. Cohen and co-workers reported the synthesis of iPS-*b*-PBD by condensation of iPS-CH(Ph)NH_2_ amine and PBD-COOH that bears a carboxylic acid chain-end [112]. iPS-CH(Ph)NH_2_ was produced by anionic polymerization of styrene initiated by *t*BuOLi/*n*BuLi at low temperature (typically −30°C) and aminated by addition of *N*-benzylidene(trimethylsilyl)-imine. Later on, two other stereoregular diblock polystyrene copolymers were synthesized by click-coupling of the two homopolymer blocks. iPS-*b*-PEG copolymer was synthesized by thiol-ene coupling of iPS-(1,7)octadiene and PEG-SH to form self-assembled materials [113]. sPS-N_3_ and PMMA- blocks were also cross-linked via Huisgen 1,3 dipolar cyclo-addition for the formation of sPS-*b*-PMMA polymer [114].

## Figures and Tables

**Table 1:** Stereoregular styrene-ethylene copolymers: catalytic system and polymer properties.

Copolymer Microstructure	Catalytic System	*T*_polym_ (°C)	Productivity (kg·mol^−1^·h^−1^)	St Incorporated (mol %)	*M*_n_ (×10^−3^ g·mol^−1^)	*M*_w_/*M*_n_	*T*_m_ (°C)	Reference
Blocky sPS-*co*-PE	**2**/BF_20_	25	600–2314	13–87	79–162	1.1–1.2	214–245 or n.o.	[27]
Random sPS-*co*-PE (*r* > 81%)	**9**	60–120	11–2529	45–97 (±2)	9–152	1.2–3.8	205–241 or n.o.	[30]
ESI with sPS block (64%–69%)	**20**/MMAO	50–90	160–900	64–80	6–58 (*M*_w_)	1.6–1.9	254–265	[33]
Block-like sPS-*co*-PE	**18**/MMAO	−20–50	0.8–70	29.2–75.5	13–2,192	1.3–2.7	252.7–264.5 (sPS part) 113.8–121.8 (PE part)	[34]
Random to blocky sPS-*co*-PE (syndiotact. > 90%)	**6a**-**6b**-**6e**-**6h**/BF_20_/TIBAL	25	1429–9571	17–78	3–94	1.2–3.0	122–272	[35]
iPS-*co*-PE	**21a**/MAO	20–50	19,300–41,400	51–99	n.r.	n.r.	n.r.	[36]

BF_20_ stands for [Ph_3_C]^+^[B(C_6_F_5_)_4_]^−^.

**Table 2:** Stereoregular styrene-propylene copolymers: catalytic system and polymer properties.

Copolymer Microstructure	Catalytic System	*T*_polym_ (°C)	Productivity (kg·mol^−1^·h^−1^)	St Incorporated (mol %)	*M*_n_ (×10^−^^3^ g·mol^−^^1^)	*M*_w_/*M*_n_	*T*_m_ (°C)	Reference
sPS-*b*-aPP	**14c**/MAO	90	450–860	n.r.	n.r.	n.r.	265–266.7	[37]
Blocky sPS-*co*-aPP	**2**/BF_20_	25	30–110	10–85	12–45	1.5–1.7	240–256 or n.o.	[39]
Blocky iPS-*co*-iPP	**21a**/MAO	0–70	18–164	66–99	2.1–60 (*M*_w_)	1.6–2.4	165/193–173/197	[41]

BF_20_ stands for [Ph_3_C]^+^[B(C_6_F_5_)_4_]^−^.

**Table 3:** Stereoregular styrene-butadiene and styrene-isoprene copolymers: catalytic system and polymer properties.

Copolymer Microstructure	Catalytic System	*T*_polym_ (°C)	Productivity (kg·mol^−1^·h^−1^)	St Incorp. (mol %)	*M*_n_ (×10^−3^ g·mol^−1^)	*M*_w_/*M*_n_	*T*_m_ (°C)	Reference
sPS-*co*-PIP	**12a**/MAO	40	0.04–0.06	7–55	n.r.	n.r.	n.r.	[42]
Blocky sPS-*co*-PBD (mix of *cis*-1,4, *trans*-1,4 and 1,2 BD insertion)	**12a**/MAO	15	8–52	2–43	31–41 (*M*_w_)	1.3–2.0	170–220 or n.o.	[43]
Blocky sPS-*co*-PBD (mix of *cis*-1,4, *trans*-1,4 and 1,2 BD insertion)	**12a**/MAO	15–70	24–36	15–92	n.r.	n.r.	205–230 or n.o.	[44]
Blocky sPS-*co*-PBD (mix of 1,4 and 1,2 insertion)	**12a-13-14b**/MAO	40	0.1–53	10–94	2–18	1.9–4.0	189–243	[45]
Random sPS-*co*-PIP (mix of 1,4 and 3,4 insertion)	**12a-13-14b**/MAO	40	0.1–35	69–97	2–19	1.9–3.2	181–250	[45]
Blocky sPS-*co*-PBD (1,4 insertion > 75%)	**19**/MAO	50	46–89	28–81	19–57 (*M*_w_)	1.9–2.4	235–254	[49]
Random sPS-*co*-PIP (1,4 insertion > 80%)	**19**/MAO	50	29–81	23–73	28–39 (*M*_w_)	1.8–2.4	Amorphous	[49]
Blocky sPS-*co*-(*cis*-1,4)PBD (*cis*-1,4 = 65%–74%)	**16**/MAO	25	4–32	22–68	10–92 (*M*_w_)	2.0–2.6	171–239	[50]
Diblock sPS-*b*-(*cis*-1,4)PBD ([*r*]^4^ > 95% and *cis*-1,4 > 70%)	**14a**/BF_20_/Al(oct)_3_	−25 (St) −40 (BD)	n.r.	n.r.	106–238	1.5–1.6	> 270	[47]
Triblock sPS-*b*-(*cis*-1,4)PBD-*b*-sPS ([*r*]^4^ > 95% and *cis*-1,4 > 70%)	**14a**/BF_20_/Al(oct)_3_	−25 (St) −40 (BD)	n.r.	ca. 85%	202	1.5	> 272	[48]
Random iPS-*co*-(*trans*-1,4)PBD	**21a**/MAO	50	8–672	15–97	6–103	1.9–2.9	Amorphous	[51]
Diblock iPS-*b*-(*trans*-1,4)PBD	**21b**/MAO	25	n.r.	30–95	139–201	1.1	42–46 (PBD) 80.4–85.9 (PBD) 222.8–224.1 (iPS)	[52]
Diblock iPS-*b*-(1,4)PBD (*trans*-1,4 = 80 mol %)	**22**/[PhNMe_2_H][BF_20_]/Al(Oct)_3_	25	n.r.	26–81	33–153	1.1–1.2	n.r.	[53]
Random or blocky iPS-*co*-(1,4)PBD	**21a**-**21c**/MAO	50	1–15	32–88	50–990 (*M*_w_)	1.7-3.7	n.o.	[54]
Diblock sPS-*b*-PIP Triblock sPS-*b*-PIP-*b*-sPS	**2**/BF_20_	25	n.r.	48–71	32–126	1.2–1.5	267–270 (sPS) −37–−35 (PIP)	[55]
Blocky sPS-*co*-PIP	**2**/BF_20_	25	9–510	2–76	34–195	1.2–2.1	189–241 or n.o.	[55]
Blocky sPS-*co*-(*cis*-1,4)PIP ([*r*]^4^ > 99% and *cis*-1,4 > 97%)	**2** + **1**/BF_20_/TIBAL	25	n.r.	34–67	28–194	1.4–1.9	267–272	[60]
Blocky sPS-*co*-(3,4)PIP ([*r*]^4^ > 99% and *3*,4 ca. 90%)	**2** + **11**/BF_20_/TIBAL	25	n.r.	50	26–67	1.7–1.8	262–263	[60]
Blocky sPS-*co*-(*cis*-1,4)PIP ([*r*]^4^ > 99% and *cis*-1,4 > 68.5%)	**8**/BF_20_	20	88–134	6–83	13–82	1.4–1.9	240–246 or n.o.	[61]
Diblock sPS-*b*-(*cis*-1,4)PIP	**8**/BF_20_	20	66	50	39	1.3	268	[61]
Diblock sPS-*b*-(*cis*-1,4)PBD ([*r*]^4^ > 99% and *cis*-1,4 > 95%)	**8**/BF_20_	20	306–396	4–85	88–225	1.3–1.7	263–268	[62]
Random sPS-*co*-(*trans*-1,4)PIP	**9**	60–80	165–1025	70–97	12–91	1.2–3.25	218–245 or n.o.	[57]
Blocky iPS-*co*-(*trans*-1,4)PIP ([*m*]^6^ = 7–98% and *trans*-1,4 > 90%)	**10**	60	2-47	30–98	59–73	2.0–2.2	51–214	[58]
Diblock iPS-*b*-(*trans*-1,4)PIP ([*m*]^6^ > 99% and *trans*-1,4 > 90%)	**10**	60	3–4	28.8–76.4	78–115	2.0–2.2	37–42; 212–219	[58]
From diblock to random sPS-*co*-PBD	**5a**-**5d**/BF_20_	15	n.r.	11.0–88.9	123–312	1.1–2.1	238–258	[64]

BF_20_ stands for [Ph_3_C]^+^[B(C_6_F_5_)_4_]^−^.

**Table 4:** Stereoregular copolymerizations of styrene with different dienes or caprolactone: catalytic system and polymer properties.

Copolymer Microstructure	Catalytic System	*T*_polym_ (°C)	Productivity (kg·mol^−1^·h^−1^)	St Incorporated (mol %)	*M*_n_ (×10^−3^ g·mol^−1^)	*M*_w_/*M*_n_	*T*_m_ (°C)	Reference
Blocky sPS-*co*-s(1,2)PPD	**12a**/MAO	−20	0.1–0.3	32–64	n.r.	n.r.	n.r.	[65]
Random PS-*co*-PPD (1,2 insertion maj)	**21a**-**21b**/MAO	50	5–200	8–85	90–620 (*M*_w_)	1.2–1.9	n.r.	[66]
Diblock iPS-*b*-i(1,2)P(4Me)PD	**21b**/MAO	25	n.r.	22–81	114–390	1.1–1.3	85–89 23–132 i(1,2)P(4M)PD 219–224 (iPS)	[67]
Alternated iPS-*co*- i(1,2)P(4Me)PD	**21b**/MAO	25	50–1000	33–95	228–968 (*M*_w_)	1.1–2.5	n.r.	[68]
Blocky sPS-*co*-PHD (*trans*-MCP: 5–72 mol %; VTM:3–14 mol %)	**4a**-**4c**/BF_20_	25	24–156	14–92	17–84	2.2–2.8	224–260	[69]
Random sPS-*co*-(1,4)PCHD	**6a**-**6j**/BF_20_/TIBAL	25	5–11	26–78	4–7	1.5–1.8	n.r.	[70]
Diblock sPS-*b*-PCL	**2**-**3**/BF_20_	r.t.	21–397	18–81	26–165	1.1–1.3	51–55 271–274	[71]

BF_20_ stands for [Ph_3_C]^+^[B(C_6_F_5_)_4_]^−^.

**Table 5:** Stereoregular styrene derivatives copolymers: catalytic system and polymer properties.

Copolymer Microstructure	Catalytic System	*T*_polym_ (°C)	Productivity (kg·mol^−1^·h^−1^)	St Incorporated (mol %)	*M*_n_ (×10^−3^ g·mol^−1^)	*M*_w_/*M*_n_	*T*_m_ (°C)	Reference
Diblock iPS-*b*-iP(*p*-Me)S	**21b**/MAO	25	n.r.	32–90	148–202	1.1–1.2	230–235	[67]
Triblock iP(*p*-Me)S-*b*-iPS-*b*-iP(*p*-Me)S	**21b**/MAO	25	n.r.	39	272	1.2	232	[67]
Diblock sP(*p*-OH)S-*b*-sP(*p*-Me)S	**14a**/BF_15_/Al(Oct)_3_	−25	n.r.	50–91	150–734	1.11–1.31	n.r.	[81]
Random sPS-*co*-sP(*p*-SMe)S	**5b**/BF_20_/TIBAL	25	n.r.	7–89	111–294	1.4–2.0	221–227 or n.o.	[83]
Diblock, triblock and quadriblock sPS-*b*-sP(*p*-SMe)S	**5b**/BF_20_/TIBAL	25	n.r.	n.r.	58 73 92	1.5 1.6 2.2	252/263/270 252/260/268 242/253/260/267	[83]
Tapered sPS-*co*-sP(*o*-OMe)S	**5d**/BF_20_/TIBAL	25	276–1220	7–90	125–147	1.1–1.3	228–266	[84]
Gradient sPS-*co*-sP(*m*-OMe)S	**5d**/BF_20_/TIBAL	25	380–1260	5–91	123–182	1.3–1.5	263-265 or n.o.	[84]
Random sPS-*co*-sP(*p*-OMe)S	**5d**/BF_20_/TIBAL	25	64–1100	4–96	69–246	1.3–2.1	213-249 or n.o.	[84]
Diblock iP(*p*-Me)S-*b*-(*trans*-1,4)PBD	**21b**/MAO	25	n.r.	33–85 (*p*Me)S	95–177	1.1–1.4	46–50/ 85–94	[67]
Random iP(*p*-Me)S-*co*-(*trans*-1,4)PBD	**21a**/MAO	25	15–827	28–93 (*p*Me)S	21–270 (*M*_w_)	1.7–2.6	n.o.	[85]
Random iP(*p*-Me)S-*co*-(*trans*-1,4)PIP	**21a**/MAO	25–50	1-117	20–92	12–81 (*M*_w_)	1.2–1.7	n.o.	[85]
Blocky sP(*p*-Me)S-*co*-(*cis*-1,4)PBD	**19**/MAO	50	41–121	19–69 (*p*Me)S	37–63 (*M*_w_)	2.8–3.8	n.o.	[85]
Blocky sP(*p*-Me)S-*co*-(*cis*-1,4)PIP	**19**/MAO	n.r.	31–58	19–68 (*p*Me)S	42–61 (*M*_w_)	1.8–2.5	n.o.	[85]
Blocky sP(DEAS)-*co*-(*cis*-1,4)PBD	**4c**/BF_20_	25	n.r.	9–97	46–106	1.3–1.4	214–249	[86]
Diblock sP(DPAS)-*b*-(*cis*-1,4)PBD	**4c**/BF_2**0**_	25	n.r.	12–51	89–115	1.9–2.0	239–241 289–293	[86]
Multiblock sPS-*co*-aPS	**17**/MAO	10–15	0.3	[*r*] = 71–86	100–140	1.3–1.4	251–262	[88]

BF_20_ and BF_15_ stand for [Ph_3_C]^+^[B(C_6_F_5_)_4_]^−^ and B(C_6_F_5_)_3_, respectively.

**Table 6:** Stereoregular styrene-based terpolymers: catalytic system and polymer properties.

Copolymer Microstructure	Catalytic System	*T*_polym_ (°C)	Productivity (kg·mol^−1^·h^−1^)	St Incorporated (mol %)	*M*_n_ (×10^−3^ g·mol^−1^)	*M*_w_/*M*_n_	*T*_m_ (°C)	Reference
Triblock iP(*p*-Me)S-*b*-iPS-*b*-iP(*p*-*t*Bu)S	**21b**/MAO	25	n.r.	40 (iP(*p*-Me)S) 42 (iPS) 18 (i(*p*-*t*Bu)S)	340	1.23	227 (iPS) 292 (iP(*p*-*t*Bu)S)	[67]
Triblock iP(*p*-Me)S-*b*-iPS-*b*-iP(4-Me)PD	**21b**/MAO	25	n.r.	39 (iP(*p*-Me)S) 38 (iPS) 22.2 (iP(4-Me)PD)	315	1.27	227 (iPS) 83 (iP(4-Me)PD)	[67]
Random sPS-*co*-PE-*co*-PP	**2**/BF_20_	25	104–930	5–32 (sPS) 43–74 (PE) 3–43 (PP)	32–153	1.1–1.8	228–229 or n.o.	[39]
sPS-*co*-PE-*co*-(*trans*-1,4)PIP	**9**	60–80	33–375	41–96 (sPS) 3–34 (PIP) 1–23 (PE)	41–83	1.2–2.8	204–219 or n.o.	[57]
Blocky sPS-*co*-(*cis*-1,4)PIP-*co*-(*cis*-1,4)PBD (ca. 75 mol % of (*cis*-1,4)PIP and 97 mol % of (*cis*-1,4)PBD)	**8**/BF_20_	20	111–144	18–36 (sPS) 23–43 (PIP) 21–40 (PBD)	48–88	1.3–1.5	242–251 or n.o.	[61]
Triblock sPS-*b*-(*cis*-1,4)PIP-*b*-(*cis*-1,4)PBD (ca. 80 mol % of (*cis*-1,4)PIP and 96 mol % of (*cis*-1,4)PBD)	**8**/BF_20_	20	46	23 (sPS) 38.5 (PIP) 38.5 (PBD)	66	1.66	265	[61]
Blocky sPS-*co*-(*cis*-1,4)PIP-*co*-(*cis*-1,4)PBD ([*r*]^4^ > 99%, (*cis*-1,4)PIP >97 mol % and (*cis*-1,4)PBD) >97 mol %)	**2** + **1**/TIBAL	r.t.	n.r.	1/1/1 sPS/PIP/PBD	94–166 (*M*_w_)	1.4–1.5	266–267	[60]
Random sPS-*co*-(1,4)PCHD-*co*-(1,4)PIP ((1,4) PCHD = 100% and (1,4) PIP >100 mol %)	**6h**-**6j**/BF_20_/TIBAL	25	5–12	6–40 (sPS) 16–63 (PIP) 31–64 (PCHD)	5-7	1.5–1.9	n.r.	[89]
Blocky sPS-*co*-PE-*co*-PHD (VTM units <1 mol %)	**4c**/BF_20_	25	925–2119	7–38 (sPS) 49–66 (PE) 12–27 (PHD)	90–128	1.3–1.5	100–120 206–243	[69]
Blocky sPS-*co*-PE-*co*-PHPD (ca. 3/1 of MCH/ECP)	**4c**/BF_20_	25	922–3698	15–83 (sPS) 9–33 (PE) 8–52 (PHPD)	38–150	1.6–2.4	247–266	[90]
Random sPS-*co*-PE-*co*-PDCPD	**2**/BF_20_	25	500–1800	4–46 (sPS) 36–69 (PE) 5–26 (PDCPD)	381–511	1.2–1.4	n.r.	[92]

BF_20_ stands for [Ph_3_C][B(C_6_F_5_)_4_].

**Table 7:** Properties of graft and diblock stereoregular styrene copolymers.

Microstructure	Styrene Content (mol %)	*M*_n_ (×10^−3^ g·mol^−1^)	*M*_w_/*M*_n_	*T*_m_ (°C)	Reference
sPS-*graft*-aPS	-	32–46	1.5–1.7	231 or n.o.	[95]
sPS-*graft*-PIP (*cis*-1,4 = 70 mol %, *trans*-1,4 = 23% and 3,4 = 7 mol %)	74–94	15–77 (*M*_w_)	n.r.	228–256	[94,96]
sPS-*graft*-iPP	-	n.r.	n.r.	217–234 (sPS) 128–130 (iPP)	[97]
sPS-*graft*-PMMA	-	n.r.	n.r.	241–253 or n.o.	[100]
sPS-*graft*-PMMA	-	n.r.	n.r.	217	[102]
sPS-*graft*-aPS	-	n.r.	n.r.	219	[102]
sPS-*graft*-PGMA	-	n.r.	n.r.	n.o.	[103]
sPS-*graft*-aPS	-	n.r.	n.r.	n.o.	[104]
sPS-*graft*-[aPS-*co*-aP(*p*-Me)S]	-	n.r.	n.r.	205	[105]
sPS-*b*-PMMA sPS-*b*-PBMA	n.r.	15–70 (sPS) 2–17 (PMMA) 7–18 (PGMA)	n.r.	n.r.	[106]
sPS-*b*-PCL	40–62	96–185	n.r.	n.r.	[107]
sP(*p*-MeR)S-*b*-PLLA	n.r.	14.8–15.8	1.2	194–224 (sP(*p*-Me)S 159 (PLLA)	[108]
sPS-*b*-aPS	20–84 (sPS)	17–51	1.9–2.4	269	[109]
iPS-*b*-PCO	4–17 (wt%)	19–39	2.9–7.4	189–222 (sPS) −2 to 0 (PCO)	[111]
iPS-*b*-PBD (>90 mol % of (1,4)PBD and > 95% of isotactic content)	66–79 (w%)	41–100 (iPS) 11–52 (PBD)	n.r.	210–216 (iPS)	[112]
iPS-*b*-PEG	n.r.	17	1.54	n.r.	[113]
sPS-*b*-PMMA	53–94	14–21	n.r.	n.r.	[114]
